# Electronic adaptation and danish cross-cultural translation of PEmb-QoL and VEINES-QoL/Sym for patients with venous thromboembolism

**DOI:** 10.1186/s41687-024-00698-9

**Published:** 2024-02-26

**Authors:** Stine Foged Lindegaard, Anette Arbjerg Højen, Nanna Rolving

**Affiliations:** 1 Danish Center for Health Services Research, Aalborg University Hospital and Aalborg University , Aalborg, Denmark; 2 Department of Physical and Occupational Therapy, Aarhus University Hospital, Palle Juul-Jensens Boulevard 99, Aarhus N, 8200 Denmark

**Keywords:** Electronic adaptation, Cross-cultural translation, Quality of life, Pulmonary embolism

## Abstract

**Purpose:**

Most patient-reported outcome (PROs) used in thrombosis research and clinical practice are delivered using technology like online questionnaires. However, only few have undergone formal electronic adaptation from paper to digital versions, threatening the validity and reliability of the PROs. The present study aimed to perform an electronic adaption and cross-cultural translation of two PROs measuring health-related quality of life in a Danish cohort of patients with venous thrombosis (VTE), specifically the VEINES-QoL/Sym questionnaire and the PEmb-QoL questionnaire.

**Methods:**

The electronic adaption and cross-cultural translation processes followed the international guidelines recommended by ISPOR. The migration of the questionnaires from paper to electronic versions was conducted in the Research Electronic Data Capture (REDCap). Following approval of the electronically adapted and translated versions, a pretest of the questionnaires was performed by cognitive interviewing patients with VTE recruited from a hospital setting.

**Results:**

Nine men and ten women between the age of 19 and 73 years participated in cognitive interviews. The questionnaires were successfully adapted from paper to electronic versions, and during the migration process only a few modifications to the content and format were made. Most comments were related to technicalities, e.g. touch functions and checkboxes. The cross-cultural translation of both questionnaires was satisfactory, as only minor rephrasing was required.

**Conclusions:**

The original and Danish version of VEINES-QoL/Sym and PEmb-QoL were successfully adapted into electronic versions and are ready to share for REDCap users. Furthermore, the Danish versions of the two questionnaires have shown satisfactory face validity.

## Introduction

Venous thromboembolism (VTE), including pulmonary embolism (PE) and deep vein thrombosis (DVT), is the third most frequent acute cardiovascular syndrome globally, following myocardial infarction and stroke [1]. The incidence rates for PE and DVT range from 39 to 115 and 53–162 per 100,000 person years, respectively [1, 2], and increase with age as incidence of VTE is almost eight times higher in individuals aged > 80 years compared to individuals in their 50’ies [2]. Although less frequent than myocardial infarction and stroke, VTE mortality ranks higher among the causes of cardiovascular mortality [2]. In addition to being frequent and serious, the frequency of negative consequences following VTE are common. The recurrence rates of VTE is reported to be as high as 36% within ten years after an initial event [3–5], and between 40 and 60% of patients experience continued symptoms like pain, cramps and swelling of the affected leg in the case of DVT, and shortness of breath, dizziness and fatigue following PE [3, 6–8]. Naturally, these sequelae have a substantial impact on the patients’ everyday life as impaired physical and mental health may limit their ability to participate in work and social activities [6, 8–12].

The clinical endpoints (such as recurrence and mortality) for people experiencing a VTE event are well known and thoroughly studied. While such endpoints are important, they are limited in their ability to describe and quantify the patients’ own experience of the impact of the VTE on their health and daily functioning. Health-related quality of life (HRQoL) define health in broader term than morbidity and mortality alone, and thereby provide valuable information about the burden of illness on the individual person’s life situation. HRQoL is therefore an important endpoint to consider when studying patients recovering from a serious disease or injury [6, 13–15].

Until recently, the mode of data collection of patient-reported outcomes (PRO), including HRQoL, has been by using paper administration [16]. E.g., patients would be required to fill out a questionnaire in connection with an outpatient visit or during hospitalization. However, with the digitalization of the health care systems in the last decade, especially in the Nordic countries, the mode of data collection of PRO-data has been changing [17, 18]. Thus, technologies that enable electronic administration of PRO-data have been developing rapidly, e.g., via computer, tablet, or handheld devices.

Several disease-specific instruments have been developed for measuring HRQoL after VTE, of which the VEnous Insufficiency Epidemiological and Economic Study (VEINES-QoL/Sym) questionnaire for DVT and the Pulmonary Embolism Quality of Life (PEmb-QoL) questionnaire for PE have been suggested as the most suited for all aspects of VTE [6, 19–21]. The questionnaires have been translated into several languages with cross-cultural adaptations showing good validity and high reliability [22–27].

When changing from one mode of data collection to another, the goal should be to have minimal or no impact on the questionnaire characteristics of the instrument of interest [16]. Thus, it should be ensured that respondents interpret and respond to the questions in the same way, regardless of the data collection mode. The ISPOR ePRO task force has developed a set of recommendations for undertaking the migration process, suggesting that this is evaluated by conducting cognitive interviews with subjects from the target population, and if needed (e.g. in the case of moderate or major changes to the electronic version), a response equivalence study may be required [28]. However, to what extent this process is performed in clinical practice and / or research is unclear, as it is rarely reported in studies using electronic data collection modes of existing PRO. Presently, a guideline-adherent migration of the VEINES-QoL/Sym or the PEmb-QoL from paper to electronic versions has not yet been performed. With an increased focus on the use of patient-reported HRQoL in research, clinical practice and the medical industry for VTE-patients, a successful adaptation from paper to electronic format therefore seems warranted. Thus, the aim of this study was to perform an electronic adaptation of the questionnaires. As the present study took place in Denmark, a cross-cultural translation of the two VTE PROs was performed concurrently, as the questionnaires had not yet been validated in a Danish setting.

## Materials and methods

**Fig. 1:**
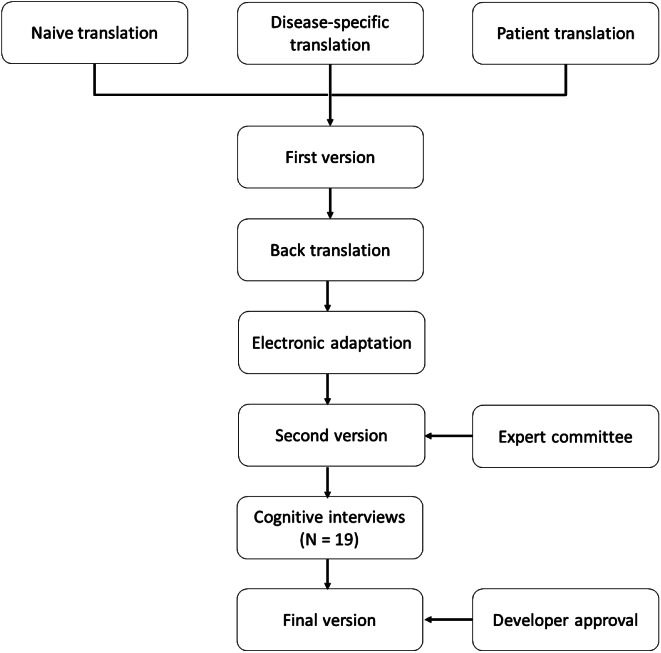
Process of the electronic adaptation and danish cross-cultural translation of the VEINES-QoL/Sym and PEmbQoL

The electronic adaptation and Danish cross-cultural translation of VEINES-QoL/Sym and PEmb-QoL followed the international guidelines recommended by ISPOR, as showed in Fig. 1 [16]. Before the migration of the questionnaires began, permission from the developer was ensured [personal communication with Susan Kahn and Erik Klok in February 2021].

### Questionnaires

The VEINES-QoL/Sym and the PEmb-QoL questionnaires are patient-reported questionnaires designed for self-completion, with content and format of the items and response scales modelled after the 36-Item Short-Form Survey (SF-36) [24, 25, 29].

The VEINES-QoL/Sym questionnaire consists of eight questions with a total of 26 items covering symptoms (Q1 and Q7, 10 items), time of day of greatest intensity (Q2, 1 item), change in symptoms over the past year (Q3, 1 item), limitations in daily activities (Q4, Q5 and Q6, 9 items), psychological impact (Q8, 5 items) [24]. Most questions cover a timeframe of the previous four weeks, and responses are rated on Likert response scales of intensity, frequency, or agreement [24]. The total VEINES-QoL/Sym summary score (25 items) provides an estimate of the overall impact of DVT on the patient’s quality of life. Q2 is not included in the summary scores [24].

The PEmb-QoL questionnaire is developed based on the VEINES-QoL/Sym questionnaire. Similar to the VEINES-QoL/Sym it measures the impact of the condition within a timeframe of the past four weeks for most questions. However, the PEmb-QoL questionnaire is distinct from the VEINES-QOL/Sym in the inclusion of PE-specific symptoms instead of DVT-specific symptoms, adding questions on limitations in daily physical activities, and extending the number of questions on emotional functioning. The PEmb-QoL questionnaire thus consists of nine questions with 40 items, covering frequency of complaints (Q1, 8 items), time of day of greatest intensity (Q2, 1 item), change in symptoms over the past year (Q3, 1 item), daily activity limitations (Q4, 13 items), work-related problems (Q5, 4 items), social limitations (Q6, 1 item), intensity of complaints (Q7 and Q8, 2 items), and emotional complaints (Q9, 10 items), [25, 29]. The total PEmb-QoL summary score (38 items) provides an estimate of the overall impact of PE on the patient’s quality of life. Q2 and Q3 is not included in the summary scores [25, 29].

For both questionnaires, higher scores reflect worse health-related quality of life. Therefore, the scale of Q1, Q4, Q5 and Q9 were reversed in the summary calculation. Item 4.a was considered missing if the answer was “*I do not work*”. As described in the initial publications [25, 29], the VEINES-QoL/Sym and the PEmb-QoL summary score were calculated by transforming all items score to a scale ranging from 0 to 100 to make items with different response scale comparable. Then an averaged of these transformed scores was calculated to obtain an overall summary score.

### Cross-cultural translation of VEINES-QoL/Sym and PEmb-QoL

The VEINES QoL/Sym and the PEmb-QoL questionnaires were cross-culturally translated into Danish, by following the systematic 5-step procedure of forward and back-translation techniques described by ISPOR [30], as shown in Fig. 1.

The forward translation was completed by three independent translators with different profiles: a naive translator, a disease-specific translator, and a patient with a history of DVT or PE. All were native Danish speakers. To support the translation process, considerations and discussions related to translated words and concepts were noted in a logbook continuously during the process (a concept elaboration document). This knowledge was used continuously during the translation process and cognitive interviews, to be able to verify that the patients had the correct interpretation of the concepts and expressions used. The translators each produced a written report of their translation, which contained comments on challenging sentences or uncertainties and rationale for their choices [31].

The three forward translations were compared in a synthesis process, including all three translators, and after consensus was reached on any discrepancies, temporary Danish versions of the questionnaires were developed (First version). The first versions of the Danish questionnaires were forwarded to a native English professional translator, who translated the questionnaires back into English. The back-translator produced a written report with comments to the translation. The back-translations were compared and discussed by those responsible for the forward translations, and if necessary, adaptions to the Danish version were made. Before field testing, an electronic migration of the first version was performed as described below (Fig. 1). The final translated version of the PEmbQoL was approved by the original developer professor F.A. Klok, while the developer of the VEINES-QoL/Sym, professor Susan R. Kahn, had approved of the overall project, but declared that no official developer approval of the final translated version was required (personal communication 25th of February 2021).

### Electronic adaption of VEINES-QoL/Sym and PEmb-QoL

Both the original English versions and the Danish versions of the questionnaires were transferred and electronically adapted into Research Electronic Data Capture (REDCap) following the steps recommended by the ISPOR ePRO task force to conduct an accurate migration of the questionnaires from paper to electronic versions [16]. The primary goal of the migration process is to ensure that patients interpret and respond to the questionnaires the same way regardless of the data collection mode [16]. Therefore, the amount of modifications to the content and format compared to the original versions should be as small as possible [16, 32].

The subsequent migration process included the identification of any necessary changes that needed to be made to suit the electronic version (Second version) including wording of instructions, choice of checkboxes, and overall layout and format (e.g. colors, font size and style, spacing, page breaks) [16, 28]. The adjusted Danish electronic versions of both the VEINES-QoL/Sym and the PEmb-QoL questionnaires were then discussed and revised by a committee of experts within the field, and a prefinal version of each questionnaire was prepared for field testing [31]. As part of the electronic adaptation, usability testing with the respondents from the target population was performed, with the purpose of examining whether respondents are able to use the software and the device appropriately [16]. This process includes formal documentation of respondents’ ability to navigate the electronic platform, follow instructions, and answer questions. Usability testing was performed concurrently with the cross-cultural translation process, i.e. was investigated and documented using cognitive interviewing [16].

### Participants

Patients were recruited from Silkeborg Regional Hospital, Aarhus University Hospital and Aalborg University Hospital in the period April 2021 to March 2022. Patients were eligible for participation if they had DVT or PE as their primary diagnosis, if they were more than one month after the VTE-event, and were native Danish speakers. Patients were excluded in case of cancer-related VTE, pregnancy, a diagnosis of anti-phosphor lipid syndrome, or if they had cognitive difficulties.

### Field testing

The field testing aimed to investigate both the cross-cultural translation of the questionnaires, as well as the different aspects of usability of the electronic version. Therefore, the prefinal versions of the electronic Danish version (second version) were tested with cognitive interviewing following ISPOR and The Three-Step Test-Interview [33] (Fig. 1). Each respondent completed one of the two questionnaires, depending whether on their primary condition was PE or DVT.

Patients were informed of the study by clinicians in the cardiologic departments at the three recruitment sites, asking for permission to forward their contact information to the project team. Patients who accepted were subsequently contacted by the primary author (SFL) by phone, who informed in more detail about the study. If the patient agreed to participate, an appointment was made in the patient’s home within a timeframe of approximately one month. Once the interviewer had arrived at the patient’s home, an email with a link to the online questionnaire was sent to the participant, from which the electronic questionnaire would automatically open in a separate browser window. Participants completed the questionnaire on a PC, tablet or smartphone. Respondents were first asked to complete the questionnaire without interruptions from the interviewer [33]. The interviewer (SFL) was seated in a position left or right of the participant, where it was possible to observe both the screen as well as the participant’s face during the entire completion process. The interviewer noted the respondents’ ability to navigate the electronic platform, follow instructions, and answer questions. Then respondents were cognitive interviewed, to probe about what the respondent thought was meant by each questionnaire item and the chosen response.

### Data analysis

Each respondent’s perception of the questionnaires and the respondents’ ability to use the electronic platform were explored to evaluate the electronic adaptation and Danish cross-cultural translation. Data from the cognitive interviews were analyzed using step-by-step guide for thematic analysis presented by Braun and Clarke (2006) [34]. Both the interviewer’s observations and the cognitive interviews were transcribed and reviewed individually and coded with individual initial codes. Then all codes across the data material were compared and thematized in categories; Layout, Technique, Q1, Q2, Q3, Q4, Q5, Q6, Q7, Q8, Q9 (only PEmb-QoL). Based on field testing, the questionnaires were adjusted, which resulted in the final version of the questionnaires (Fig. 1). The original and the Danish electronic versions of the VEINES-QoL/Sym and the Pemb-QoL is available in REDCap and available by contacting the main author.

### Ethical considerations

The study was conducted in accordance with the Helsinki Declaration [35]. In accordance with Danish law, interview studies do not require approval from a Health Research Ethics Committee (the Act on Research Ethics Review of Health Research Projects (§ 14, subsection 2)). The patients all signed a written consent form before entering the study and were informed that their consent could be withdrawn at any time point and without consequences. All data were stored and managed confidentially on secure servers in Central Denmark Region [journal no. 1-16-02-393-22] and North Denmark Region [journal no. 2021-006], respectively, in accordance with the European General Data Protection Rules.

## Results

A total of 19 patients with a history of VTE pretested one of the two questionnaires, depending on their primary condition (DVT or PE). Purposive sampling was used to ensure sufficient diversity in age, sex, and VTE condition. Patients’ characteristics are shown in Table 1. Ten patients with DVT completed the VEINES-QoL/sym questionnaire and nine patients with PE completed the Pemb-QoL questionnaire. Most of the patients answered the questionnaire using their computer or smartphone.

**Table 1:** Characteristics of the 19 patients included in the study

Characteristics	
Age, mean (range)	48.52 (19–73)
Sex, n (%) Men Women	9 (47.37) 10 (52.63)
Diagnose, n (%) PE DVT	9 (47.37) 10 (52.63)
Answer devices, n (%) PC Tablet Smartphone	11 (57.89) 3 (15.79) 5 (26.32)

### Cross-cultural translation

In order to maintain the meaning of the original questions, the following changes were made to translate and adapt the questionnaires into Danish. A complete overview of changes made is shown in Table 2. The title of both questionnaires was changed, as the patients could not understand the titles as they were. Furthermore, instead of the expression “*leg problem*” appearing several times in the introductory text of the VEINES-QoL/Sym questionnaire, the Danish equivalent of “*blood clot in the leg*” was used, based on recommendations from the translator group. For the remaining questionnaire, the Danish translation of “*problem with your leg*” was used throughout the questionnaire. Item 2.e was translated to “*At any time of the day*”, but during cognitive interviews patients answered that they have intense symptoms at several times of the day, but not at all times of the day. Therefore, they want to check off more than one answer (e.g., both “*on waking*” and “*during the night*”), which was not possible. Therefore, the item was changed to “*At several times of the day*”. In item 4.c, “*Taking public transport*” is mostly not associated with a standing activity in Denmark, but cognitive interviewed patients answered that they have problems standing in queues for long time, therefore the wording was changed to “*standing in long queues*”. In Q6, “*your normal social activities*” was changed to “*your social activities*”, since normal social activities can be misunderstood in a Danish context. In the response categories of Q4 and Q5 we changed the words from uppercase to lowercase, to comply with Danish orthography (e.g. corresponding to YES, Limited A Lot, changed to Yes, limited a lot). In addition, bold and underlines were standardized to make the questionnaires more manageable for the respondents. In Danish, words related to time are often placed differently in sentences, therefore time phrase *“…during the past 4 weeks?”* in Q7 and Q8 was placed in front instead of at the end of the sentence.

**Table 2:** Adjustments made to the electronic danish version of PEmb-QoL and VEINES-QoL/Sym questionnaire from 19 cognitive interviews with patients who have had a VTE

	Problem	Explanation	Changed to
**Translation process**	Title wording “VEINES-QoL/sym questionnaire” and “Pemb-QoL questionnaire”	Patients did not understand the content and purpose of the questionnaires based on the title	“VEINES-QoL/sym questionnaire on quality of life after venous thrombosis” and “Pemb-QoL questionnaire on quality of life after pulmonary embolism”
	In item 2.e, the wording “At any time of the day” understood as “continuously”	Patients experiencing symptoms at several times of the day, but not at all times of the day, wanted to check off more than one answer, which is not possible.	“At several times of the day”
	In item 3 “…, how would you rate the condition of your lungs/leg problem in general now?”	Patients questioned the term “in general”, so we removed it.	“…, how would you rate the condition of your lungs/leg problem now?”
	In item 4.c in VEINES-QoL/sym, the wording “taking public transportation” not associated with standing for a long time	Taking public transport is mostly not associated with a standing activity in Denmark, but patients do have problems standing in long queues.	“Standing in long queues”
	Phrasing of questions in items 5a– 5d	The wording of questions was changed from passive to active form.	E.g. 5a. “I have cut down the amount of time…”
	In item 6 the word “normal” in the sentence “…your normal social activities…”	“Normal activities” could be misunderstood in Danish, i.e., there may be a difference between what society means are normal activities and what your normal activities are.	“…your social activities…”
	In Item 7a-7f and item 8a-8f the response items were specified.	“Pain” was added to all items in question 7 and “breathless” was added to all items in question 8.	E.g. question 7 a.) No pain
	In item 7 and 8, the position of the time phrase “…during the past 4 weeks?”	In Danish, words related to time are often placed differently in sentences compared to English.	“During the past 4 weeks, how…”
	In item 9. i., the wording “traveling” was only associated with longer journeys, e.g. going abroad.	The word “taking a trip” was added since patients do not travel that much, but rather go on smaller trips where they may be limited due to their VTE event.	“…feel limited in traveling or taking a trip?”
**Migration process**	**Problem**	**Explanation**	**Change**
	Patients skip the introduction text and go directly to the questions.	Because touch-screen tablet and mobile phone offers less screen space, introductory texts were too extensive, and patients scroll directly to the questions.	Introductory texts were reduced and simplified.
	Response buttons were too small	Patients found it difficult to click on response buttons both on touch-screen and computers because the buttons were too small.	Response buttons were enlarged where possible in REDCap settings.
	Depending on the device used by patients, word division was sometimes wrong in sentences.	Because computers and some tablets offer more screen space, the text is presented in larger font and displayed on the same screen automatically. Smaller devices have more compact text, and the word division were sometimes wrong.	Front sizes were changed from automatically size to a permanently size, and manual word division were added.

### Electronic adaption process

**Fig. 2:**
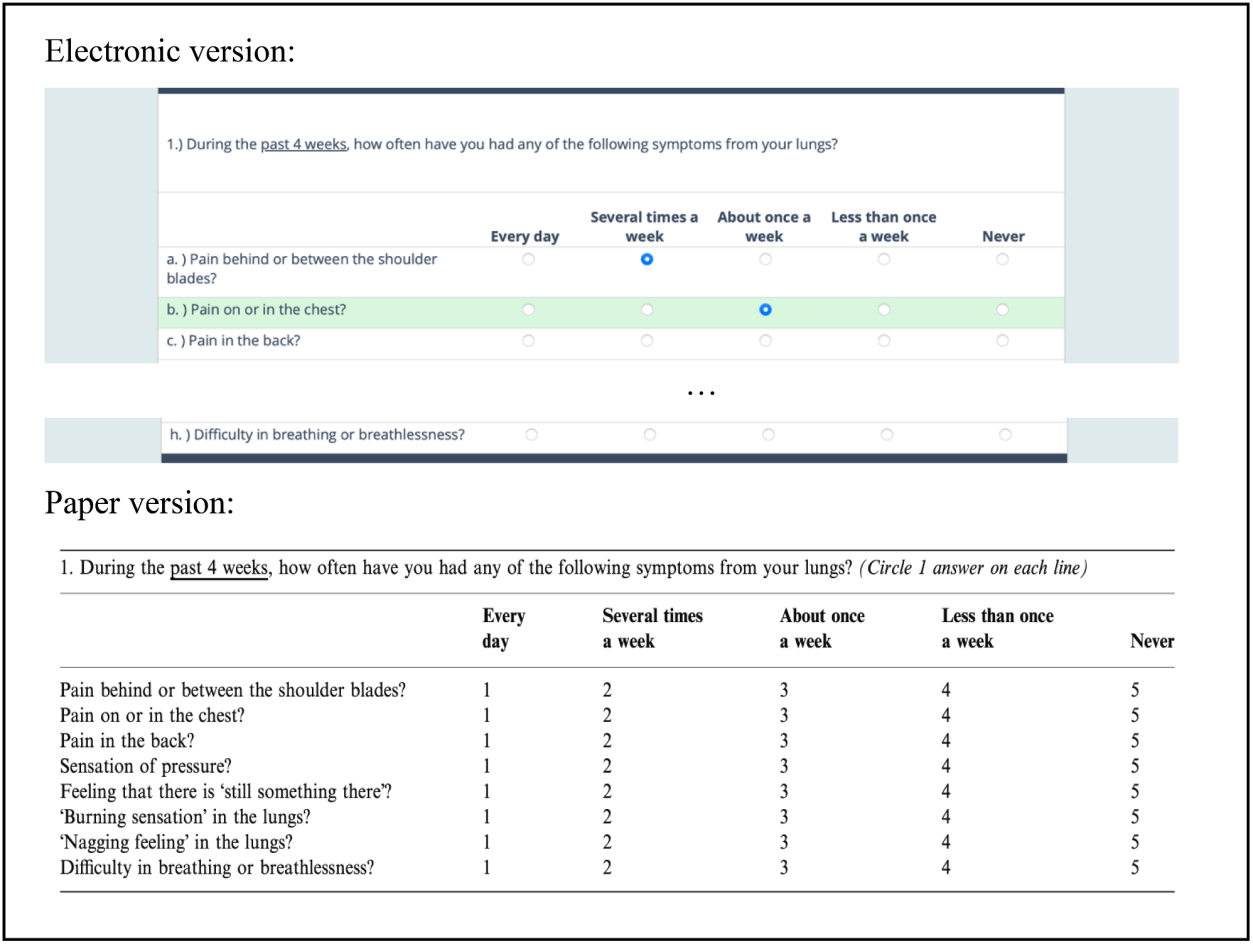
Example of electonic version versus paper version in question 1 of PEmbQoL

Overall, the paper version of the questionnaires was suitable for an electronic format, and most questions were directly inserted in REDCap. The adaptation process primarily required changes related to layout considerations like font size, section divisions, and spacing between questions and responses. Furthermore, equal spacing of response options was ensured to reduce bias and improve usability. Challenges included REDCap limitations in design and layout options, especially the response buttons in matrixes. Even though the cognitive interview showed that patients found it difficult to click on response buttons both on touchscreen and computers because the buttons were too small, it was not possible to change the size of the buttons. Patients reported that they prefer to scroll down on their electronic device to see the next questions instead of switching page. Therefore, the questionnaires were organized continuers. At cognitive interviews, some patients skipped the introductory text and went directly to the questions. When asked, the patients replied that the text was too extensive on a small screen and therefore the introductory text were reduced and simplified. In the electronic format by default, the patients cannot finish the questionnaire, if the answers are missing. To align with the paper version providing the patients with the opportunity not to answer a given question, the setting was changed to allow missing answers and a new button*” Are you sure you do not want to answer all questions?”* were added to the end of the questionnaires to ensure missing answers were deliberate. If the Patients answer “*Yes*” they can finish without answering all questions, and if they answer “*No*” the missing response is highlighted. An example of paper and digital versions of questions and response options is shown in Fig. 2. A detailed description of the changes required during the electronic adaptation process is shown in Table 2.

## Discussion

The VEINES-QoL/Sym and PEmb-QoL were originally developed for paper administration. These have now been successfully adapted for electronic administration and are ready to share for REDCap users [36]. The two questionnaires are based on the SF-36 Health Survey, and therefore the electronic adaptions performed in this article, can be compared to existing literature on electronic adaption of the SF-36. The SF-36 is a widely used PRO measuring generic health-related quality of life [37, 38], and several electronic versions of SF-36 have been published [39]. A meta-analysis, including 25 studies, reviewing measurement equivalence of the SF-36 across electronic versus paper administrations, showed good equivalence across a variety of disease populations, languages, and electronic modes [40]. This supports our finding, that migrating the VEINES-QoL/Sym and Pemb-QoL from paper to electronic mode of administration does not substantially alter the way in which participants respond to questionnaires. This also aligns with the ISPOR guidelines for electronic migration, suggesting that equivalence studies of electronic versus paper versions are only required in the case of major changes in the electronic adaptation process [16].

Regarding the cross-cultural translation process, the Danish versions of the two VTE PROs showed satisfactory face validity and only a few changes from the original version were made in the translation process. Cross-cultural validation of the two questionnaires for other languages, e.g. Norwegian, Swedish, Dutch and French, have similarly shown that the psychometric properties are comparable with the original English version [22, 23, 27, 41]. Seeing that Denmark belongs to a Western European culture, it is therefore reasonable to assume, that the Danish versions of the VEINES-QoL/Sym and PEmb-QoL are valid HRQoL instrument for VTE.

It is recommended that PRO questionnaires should be as brief as possible in order to minimize the respondent’s burden [42]. This recommendation is consistent with findings from this study’s cognitive interviewing, where some patients skipped extensive text passages and went straight to the questions. By minimizing and specifying the introductory text, patients found it easier to read and respond to the questions. In addition, the wording and sentence structure were also changed to minimize the response burden in other questions in the questionnaires. The fact, that ePROs offer less space than paper PROs due to the smaller screen also argues for a simplification of the introductory text and questions, so the need for scrolling up and down the screen is minimized. However, when simplifying questions, we were aware that too much simplifying could result in misunderstandings. The meaning and understanding of all questions were ensured in cognitive interviews, where all patients were asked about what they thought was meant by each questionnaire item and the chosen response observation.

When migrating a survey from one mode to another, aspects of formatting, layout, and even text size may differ between modes [28]. In the present study, the setup of the electronic versions of the VTE PROs was similar to the paper versions, but the ePROs were in some questions challenged by REDCaps limited design and layout options (see Fig. 2; Table 2). The layout differed depending on the size of the electronic device screen and participants pointed out that the response buttons were too small, especially in matrixes, for all electronic devices. Previously published meta-analyses of PRO mode equivalence reported that this may affect the user-friendliness, the patients’ willingness to respond, or even the patients’ ability to respond (i.e., in visually impaired) [43, 44]. In the present study we tested the questionnaires on different electronic device screen (smartphone, tablet, computer and both Windows Android and IOS) to ensure that the layout and other functionalities worked perfectly on every device.

Another consideration, when implementing electronic modes of administration in health care, is the issue of e-health literacy and inequality in health. Thus, it is important to consider the consequences for the representativeness of the target group. The cognitive interviews in this article showed that older people were more technically challenged, and it took them longer time to complete. They found it difficult both to open the questionnaire via the link in the email, and to click the buttons in the questionnaire, and some needed help from their relatives. VTE affects a wide range of people, but the majority of patients affected by VTE are older. It should therefore be considered whether it is appropriate to send the questionnaires electronically to all patients, as it may affect the response rate and thereby treatment choices and access to care. Also, we have to deal with the fact that some people do not have an electronic device, internet access or access to digital mailbox, or simply find it challenging to use e-health systems. Therefore, people who would otherwise be able to complete the questionnaire in paper will be excluded from answering.

### Strength and limitations

The study has several strengths. First, the study adheres with the ISPOR guidelines for cross-cultural and conceptual translation and electronic adaptation of the VEINES-QoL/Sym and the PEmb-QoL [16]. Of note, the ISPOR guidelines were published almost two decades ago, and the recommendations brought forward in a more recent publication by McKown and colleagues could profitably be considered in future studies concerned with cross-cultural translation and adaption of PROMs [45]. The study was, however, not published at the time our study was initiated. Second, the migrated Danish version underwent cognitive testing in a population of both young, middle-aged, and older patients of both genders to ensure representativity of the target group. Third, the study was strengthened by the choice of conducting the cognitive interviewing in the patient’s own home using their own electronic device, to best reflect a real-life situation.

The choice of using REDCap for the electronic adaptation could be considered a limitation of our study. Although REDCap is freely available and is presently being used by more than 6,000 institutions worldwide [36], using the database requires access, as well as some extent of technical knowledge and skills.

### Conclusion and perspectives

With the increased focus on providing evidence–based care throughout our healthcare systems, the use of valid questionnaire is essential if we want to be able to trust our assessment of a patient’s condition, monitor progression or decline, and the effects of our interventions. At the same time, the increased digitization of the healthcare system requires PROs to be accessible electronically. In this article the Danish version of the VEINES-QoL/Sym and the PEmb-QoL was successfully translated and electronically adapted into REDCap and can now be used electronically in both practice and research in a Danish setting. Overall, the two questionnaires showed satisfactory face validity and only a few changes from the original version were made in the migration process.

## Data Availability

Not data as such was generated during the study, but the qualitative interview data (in Danish), supporting the findings of our study, are available upon reasonable request to the corresponding author.
